# The Influence of Gaming Behavior on School Adjustment among Korean Adolescents: The Moderating Effect of Self-Regulation

**DOI:** 10.3390/bs14030259

**Published:** 2024-03-21

**Authors:** Jisue Lee, Goo-Churl Jeong

**Affiliations:** 1 Department of Counseling Psychology, Graduate School, Sahmyook University, Seoul 01795, Republic of Korea; a01041995661@gmail.com; 2 Department of Counseling Psychology, College of Health and Welfare, Sahmyook University, Seoul 01795, Republic of Korea

**Keywords:** game, game addiction, school adjustment, self-regulation, self-control, adolescent

## Abstract

With the rise of digital devices, gaming has become both a pastime and part of the culture for young people. Teenagers use games to communicate, enjoy leisure time, and relieve stress. However, the maladaptive use of gaming can lead to difficulties in adolescents’ daily lives and school adjustment. Increasing adolescents’ self-regulation competencies can improve maladaptive gaming behaviors and help them use gaming adaptively. Therefore, this study examined the moderating effect of self-regulation on the impact of adolescent gaming behavior on school adjustment. This study considered 359 adolescent participants in South Korea. Data were analyzed using hierarchical regression to test the moderating effect. The results indicated that adolescents’ adaptive use of games significantly increased school adjustment. Self-regulation significantly moderated the negative effects of the maladaptive use of games on school adjustment. Furthermore, the results revealed that the groups with highly adaptive and maladaptive use of games had high school adjustment but low self-regulation, indicating that they required active intervention.

## 1. Introduction

### 1.1. Gaming Culture and Issues among Korean Adolescents

In the modern world, the Internet and smart devices have grown by leaps and bounds, and almost every generation has access to digital media, which is closely connected to most aspects of daily life. New lifestyles are formed, as the dynamic environment adds new features constantly. The use of various media devices such as smartphones, computers, and tablets has become a significant part of daily lives, particularly for curious young people. According to the 2020 Youth Statistics, gaming and browsing the Internet are the most popular activities for Korean youth because of stress in their leisure time [1]. For Korean youth, gaming on media devices is already part of their culture and a way of communication.

With the development of media, Korean teenagers are forming a new culture by interacting with friends in gaming spaces. The stress of college entrance exams for adolescents has already started in childhood, and urban development has reduced the space for adolescents to play outside the home [2]. Korean adolescents had a culture of going to PC rooms in groups to play games together, and they felt good and happy when they won games or leveled up their game characters [3]. However, due to the COVID-19 pandemic, youths’ collective leisure activities have been restricted to their homes [4], and Internet games have become a great alternative activity for them. The development of media and the COVID-19 pandemic helped adolescents to form a leisure culture at home, and their gaming behavior became a natural way of daily play and communication [5].

However, adolescents in Korea are experiencing extreme academic stress due to the realistic challenges of college entrance exams [6]. There is a phenomenon that adolescents use games to avoid extreme academic stress, and conversely, they use games to endure extreme academic stress.

According to the Comprehensive Survey of Child and Adolescent Gaming Behavior (2022) [7], 11.9% of those surveyed were adaptive gamers, 3.5% were problematic gamers, 67.3% were regular gamers, and 17.3% were non-users. Adaptive gamers reported lower academic stress and higher life satisfaction than non-users. Previous studies have reported that adolescents play games primarily to relieve stress [8]. This indicates that adolescents perceive gaming as a leisure activity and use it to cope with stress.

### 1.2. Negative and Positive Functions of Gaming Behavior

Early research on gaming has largely been dysfunctional and focused on its problematic outcomes. Nevertheless, recent research on gaming is increasingly adopting a net functional approach owing to changes in social and environmental systems. Currently, there are limitations to understanding adolescents’ gaming behavior by simply dichotomizing it into negative and positive functions based on outcomes. In gaming behavior, over-immersion and adaptive use can coexist simultaneously [9], and it is necessary to conduct research with the understanding that it is possible to have both positive and negative experiences [10].

Research on gaming behavior from a dysfunctional perspective has focused on the negative consequences of over-engagement. Previous studies have indicated that excessive gaming can lead to attention deficit hyperactivity disorder (ADHD) symptoms in elementary school students [11], depression, and other health-related problems [12,13,14,15,16,17]; interfere with academic achievement [11,18,19]; and increase the tendency to refuse to go to school [20]. The fact that previous studies have attempted to identify the relationship between adolescent gaming behavior and school adjustment is partly owing to adults’ uncomfortable view that gaming behavior infringes on students’ right to study [21].

However, in recent years, there has been a steady increase in research on the net functional approach to gaming behavior, in addition to a dysfunctional perspective. In particular, studies on the net function of gaming behavior have reported positive psychological traits in adolescents. Playing games positively affect subjective well-being [22,23,24,25] and improve future well-being [26]. Adolescents who lack community experiences in the real world experience positive community experiences through online games [27], and leadership experiences in games help improve leadership in the real world [28]. This suggests that adolescents who lack a variety of experiences can experience positive elements through games that can help them feel positive and grow up in real life. Despite this growing body of positive research on gaming behavior, a vast majority of studies remain dominated by the view that gaming behavior is problematic and needs to be addressed. This problem-focused perspective can lead to a negative perception of youth gaming behavior as if some youth’s gaming addiction is representative of all youth gaming behavior. Consequently, some researchers predict that gaming behavior itself will have negative consequences for adolescent development and adjustment [12,13,14,15,16,17,18,19,20].

### 1.3. Gaming Behavior and School Adjustment

While adolescent development and adjustment can occur in any setting, including adolescents’ home, school, and society, several researchers have emphasized the importance of schools, where adolescents spend most of their time interacting daily. Schooling and adjustment are important tasks for adolescents as they form a sense of identity and prepare for adulthood. Therefore, school adjustment in adolescence should be understood in a comprehensive sense that includes not only grades but also social, psychological, and emotional adjustment [29]. Several studies were conducted to predict adolescents’ school adjustment. Numerous studies were conducted on adolescents’ psychological variables such as self-esteem [30], self-efficacy [31,32], stress coping [33], and family-related studies [32,34,35,36,37,38]. Moreover, there are studies on bullying [39] and negative gaming behaviors such as game over-engagement [16,40,41] in relation to school life. Previous studies on gaming among adolescents have shown that when adolescents are overly immersed in gaming, their academic performance decreases [42], they tend to be depressed and refuse to attend school [20], have more conflicts with their parents [43], and report sleep deprivation [44]. The rapid spread of smart devices among adolescents, owing to the development of the Internet and smartphones, has increased their gaming behavior, and it is necessary to study their effects on school adjustment. In particular, ensuring that teens can self-regulate their gaming behavior to stay on track in school is essential for guiding teen gaming behaviors. To this end, we aimed to examine how adolescents’ self-regulatory competence moderates the relationship between gaming behavior and school adjustment.

### 1.4. Gaming Behavior and Self-Regulation

Given the context of Korean adolescents’ college admissions, studies on adolescent happiness have shown that higher academic performance increases happiness [45], but there are also reports that appropriate gaming behaviors can help relieve stress and that gaming behaviors themselves increase happiness [25]. Thus, appropriate gaming behavior is a positive factor in reducing academic stress among adolescents.

Self-regulation refers to an individual’s regulatory activities that apply across a wide range of situations, including motivational, cognitive, emotional, and behavioral regulation [46]. In other words, self-regulation is the ability to transform one’s internal impulses into acceptable social forms [47] and actively regulate one’s behavior, thoughts, and emotions in response to external stimuli to achieve personal goals [48]. It is an important protective factor that helps young people take control of their own behavior and avoid the consequences of risky behavior. However, it is not always easy for adolescents to stop the pleasurable behavior of gaming on their own. Self-regulation theory suggests that self-regulation occurs when natural desires for pleasure, such as gaming behavior, come into conflict with cultural desires, such as school adjustment or grades [49,50]. In other words, when natural and cultural demands collide, a conflict of motivation arises, and the phenomenon of regulating one’s demands occurs. For Korean adolescents, gaming behaviors are very enjoyable, but the cultural pressure to fit in well in school, especially when it comes to college entrance exams, requires them to moderate their gaming behaviors. This does not mean not playing games at all but rather utilizing them in a positive way and controlling the time spent on them.

Previous studies on adolescents’ school adjustment and self-regulation have reported that self-regulation is an important psychological characteristic that helps adolescents adjust to school [51,52,53]. Additionally, self-regulation plays an important role in helping children and adolescents develop positive self-concepts by enhancing their self-esteem [54]. Low self-regulation in adolescents can lead to unstable emotions, poor academic performance, and problematic behaviors, such as bullying and Internet addiction [55,56]. Moreover, self-control is an important variable related to addiction and is closely related to behavioral addictions, such as Internet and smartphone addictions [57]. Therefore, self-regulation is an important predictor of gaming addiction in adolescents. In particular, adolescents may be more susceptible to excessive game use and have less ability to control it themselves because they may have less self-regulation and control over their environment than adults [58,59]. As problematic gaming behaviors among Korean adolescents are highly related to self-regulation [60], it is imperative to study the relationship between self-regulation and gaming behaviors among adolescents. Based on previous studies, the self-regulation of adolescents’ gaming behavior is expected to reduce the likelihood of triggering problematic gaming behavior. Therefore, this study examined the moderating effect of self-regulation on the impact of adolescent gaming behavior on school adjustment.

The hypotheses of this study are as follows:

**Hypothesis** **1.** *Adolescents’ adaptive use of games positively affects school adjustment.*

**Hypothesis** **2.** *Adolescents’ maladaptive use of games negatively affects school adjustment.*

**Hypothesis** **3.** *School adaptation differs according to the type of adolescents’ gaming behavior.*

**Hypothesis** **4.** *Self-regulation has a moderating effect on the impact of adolescents’ gaming behavior on school adjustment.*

## 2. Materials and Methods

### 2.1. Participants

We surveyed 400 students from the second year of middle school to the second year of high school in South Korea. Participants were sampled nationally by a professional survey company and were stratified by grade level and gender. From the collected questionnaires, we excluded 41 students who did not play games and analyzed the data of 359 students. Of those participants, 54.3% were boys, and 45.7% were girls, with a mean age of 15.5 years (SD = 1.1). Overall, 51.0% were middle school students, and 49.0% were high school students. On a typical weekday, 28.4% of teenagers played for less than an hour, 28.4% played for 1–2 h, and 43.2% played for more than 2 h. When categorized by gaming behavior, 53.8% of teenagers were in the moderate-use group, 37.6% in the adaptive-use group, 6.4% in the risky-use group, and 2.2% in the problematic-use group.

### 2.2. Procedure

We obtained the approval of the Institutional Review Board for the ethical conduct of this study (IRB NO. SYU 2021-11-015-006). The survey was conducted by an online survey company. Study participants were selected using quasi-proportional sampling to resemble the population composition of South Korea. The survey period was one week, starting from 27 April to 3 May 2023. An anonymous survey of adolescents was conducted. The online survey was designed such that the participants were first presented with a description of the study and asked to read it fully. After reading the study description, the online survey was designed to begin only if the students and parents agreed to participate voluntarily. The study description explained that no personally identifiable information would be collected from the participants and that the data collected would be used for research purposes only and would not be provided to third parties. Participants were also informed that if they felt tired or uncomfortable while answering the questionnaire, they could stop at any time and would not be penalized. The participants received online points from the survey organization as compensation for the time they spent answering the questionnaire.

### 2.3. Instrument

#### 2.3.1. Gaming Behavior

Gaming behavior was measured using the Composite Scale for Gaming Behavior Revised (CSG-β) [61]. The CSG-β scale consists of two instruments, the Adaptive Game Use Scale (AGUS) and the Maladaptive Game Use Scale (MGUS), which are used to diagnose gaming behavior types. It is suitable for this study because it can identify both the positive and negative effects of gaming.

The AGUS has seven subscales: fun and energizing experiences (“Playing games generates energy for enjoyable living.”); expanding life experiences (“New ideas usable in my life arise through gaming.”); managing emotions and stress (“Stress is relieved while playing games.”); immersive experiences (“I experience being completely absorbed in something while playing games.”); challenges and achievements (“I feel great when I play games well.”); feelings of control (“I learn ways to control impulses through gaming.”); and social interactions (“I make friends with whom I connect emotionally while playing games.”).

The MGUS has seven subscales: tolerance (“To feel the desired satisfaction, I need to play games much longer than before.”); withdrawal (“I feel restless and anxious if I cannot play games or reduce playing suddenly.”); excessive time spent (“I play games much longer or more frequently than planned each time.”); impaired control (“I have tried several times to reduce gaming time but failed repeatedly.”); compulsive use (“I think about games for most of the day.”); neglect of daily life (“Due to games, I miss or do not participate properly in important family activities.”); and continued use despite side effects (“Even with family conflicts, I continue playing games.”).

Each scale consists of 21 items, with 3 items for each subfactor. Each item was measured on a 4-point Likert scale. According to the scoring method proposed by previous studies, if at least three of the seven subfactors of the AGUS have a score of six or higher, they are classified as the “high group”, and the rest are classified as the “low group”. Similarly, the MGUS is also categorized into the “high group” and the “low group”. Then, considering both the AGUS and MGUS, we categorized the “adaptive-use group” as the “high group of AGUS” and “low group of MGUS”; the “general-use group” as the “low group of AGUS” and “low group of MGUS”; the “risky-use group” as the “high group of AGUS” and “high group of MGUS”; and the “problematic-use group” as the “low group of AGUS” and “high group of MGUS”. In this study, the Cronbach’s α value of the AGUS was 0.96, and the Cronbach’s α value of the MGUS was 0.95.

#### 2.3.2. Self-Regulation

Self-regulatory competence was measured using the Korean version of the Adolescent Self-Regulation Scale developed by Moilanen (2007) [62,63]. The self-regulation scale consists of 17 items, and the subscales are activation (“I can continue a task when there is a significant challenge.”); inhibition (“It is difficult for me to concentrate on long-term goals due to minor problems.”); and self-reflection (“Even when stressed, I am well aware of many events happening around me.”). The scale is answered on a 5-point Likert scale, with higher total scores indicating greater self-regulation. In this study, the scale had a Cronbach’s α value of 0.80.

#### 2.3.3. School Adjustment

To measure adolescents’ school adjustment, we used the School Adjustment Scale developed through the Academic Motivation Standardization Study [64]. The scale is composed of 20 items, and the subfactors are relationships with teachers (“I can freely converse with teachers.”); peers (“I have many friends with whom I can openly talk at school.”); school classes (“I understand most of the content learned during class time.”); and school rules (“I do not casually discard tissues or trash.”). The scale is answered on a 6-point Likert scale, with higher scores indicating better school adjustment. In this study, the Cronbach’s α value was 0.89.

### 2.4. Data Analysis

We analyzed the collected data using IBM SPSS Statistics for Windows (version 25.0; IBM Corp., Armonk, NY, USA). We present the descriptive statistics of the study variables and Cronbach’s α coefficients of the scales. Differences in self-regulation and school adjustment according to the type of gaming behavior were analyzed using analysis of variance (ANOVA), and post hoc tests were analyzed by the Tukey honest significant difference (HSD) method. The Pearson correlation coefficient was used between the research variables, and the moderating effect was analyzed using hierarchical regression analysis and the PROCESS Macro (Model 1).

## 3. Results

The correlation coefficients between gaming behavior, self-regulation, and school adjustment are presented in Table 1. We found that the adaptive use of games was significantly and positively correlated with maladaptive use (r = 0.40, *p* < 0.001), self-regulation (r = 0.15, *p* < 0.01), and school adjustment (r = 0.23, *p* < 0.001). The maladaptive use of games was significantly and negatively correlated with self-regulation (r = −0.31, *p* < 0.001) and school adjustment (r = −0.23, *p* < 0.001). Self-regulation was significantly and positively correlated with school adjustment (r = 0.54, *p* < 0.001). The descriptive statistics of the variables are listed in Table 1.

To test this more specifically, we conducted an analysis of variance that categorized gaming behavior into four groups based on the adaptive and maladaptive uses of games. First, the adaptive-use group (high adaptive use and low maladaptive use); second, the general-use group (low adaptive use and low maladaptive use); third, the risky-use group (high adaptive use and high maladaptive use); and fourth, the problematic-use group (low adaptive and high maladaptive use).

We conducted an ANOVA to test the differences in self-regulation and school adjustment between the gaming behavior groups, and the results are presented in Table 2. Post hoc tests were performed using the Tukey HSD. The ANOVA results indicated a significant difference in self-regulation between the gaming behavior groups (F = 8.75, *p* < 0.001). Post hoc tests revealed that the adaptive-use group had significantly higher self-regulation than all other groups, and the problematic-use group had significantly lower self-regulation than the adaptive-use and general-use groups.

Differences in school adjustment by gaming behavior group were also statistically significant (F = 9.50, *p* < 0.001). Post hoc tests indicated that the problematic-use group was significantly lower than all other groups. Interestingly, the risky-use group did not exhibit a significant difference in school adjustment compared to the adaptive- and general-use groups. These results account for the interaction effect between gaming behaviors.

**Figure 1:**
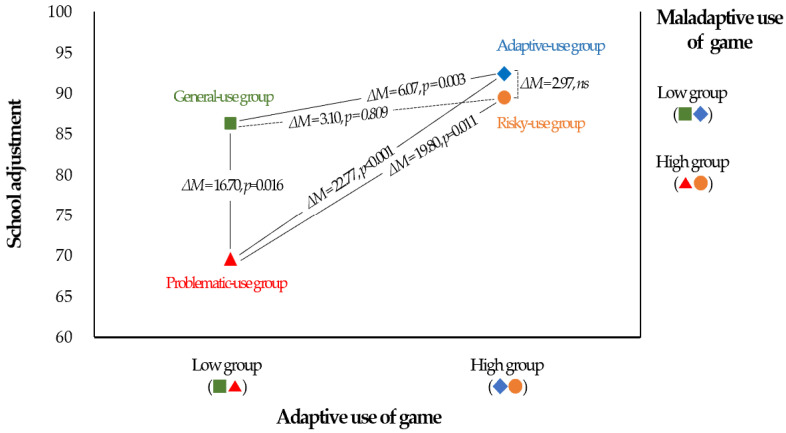
Results of the Tukey HSD test for school adjustment by gaming behavior type.

The results of the Tukey HSD post hoc test are depicted in Figure 1, along with the mean values of the gaming behavior group. In the two groups with the low maladaptive use of games (adaptive-use and general-use groups), school adaptation was very high. The highest school adjustment score was obtained when high adaptive use occurred together with low maladaptive use (the adaptive-use group). However, even if the maladaptive use of the game was high, not all exhibited low school adaptation, confirming the interaction effect between gaming behaviors. In other words, school adjustment was high in cases where the maladaptive use of games was high, but adaptive use was also high (the risky-use group). However, the lowest school adjustment is observed in the case of low adaptive use and high maladaptive use (the problematic-use group). Although the maladaptive use of games was high, school adjustment was very high when the adaptive use of games was also high. There was no statistically significant difference between the risk-use group and the normal- or adaptive-use groups.

Although the risky-use group did not differ significantly from the adaptive-use group in school adjustment, notably, the risky-use group scored significantly lower than the adaptive-use group in self-regulation.

The moderating effects of self-regulation were analyzed using hierarchical regression and the SPSS PROCESS (version 4.2) module. After controlling for the basic demographic characteristics of gender and age, the independent variables—the adaptive and maladaptive use of games—were entered using reference coding (low = 0, high = 1). The self-regulation variables were mean-centered and multiplied by the independent variables to form an interaction term.

The Durbin–Watson value of the final regression model (Model 3 in Table 3) was 1.98, indicating that the error terms were independent. The variance inflation factor (VIF) of the final regression model ranged from 1.04 to 1.88, indicating no problems with multicollinearity. The final regression model indicated a statistically significant (R^2^ = 0.322, F = 23.85, *p* < 0.001) total explanatory power of 32.2%.

In regression Model 1, where we entered the adaptive and maladaptive use of games after controlling for gender and age, the adaptive use of games had a positive effect on school adjustment (B = 7.40, *p* < 0.001), while the maladaptive use of games negatively affected school adjustment (B = −6.54, *p* = 0.019). In regression Model 2, where self-regulation was added, the adaptive use of games had a significant positive effect on school adjustment (B = 4.74, *p* = 0.001), the maladaptive use of games did not have a statistically significant effect (B = −0.81, *p* = 0.741), and self-regulation had a significant positive effect on school adjustment (B = 0.83, *p* < 0.001). In regression Model 3, in which interaction effects were added, the adaptive use of games (B = 4.39, *p* = 0.002) and self-regulation (B = 0.72, *p* < 0.001) had significant positive effects on school adjustment. However, maladaptive use had no statistically significant effect (B = 2.78, *p* = 0.343) when combined with self-regulation. In addition, looking at the interaction term, the moderating effect of self-regulation was not significant in the relationship between the adaptive use of games and school adjustment (B = 0.17, *p* = 0.247). However, self-regulation had a significant moderating effect on the relationship between the maladaptive use of games and school adjustment (B = 0.66, *p* = 0.035).

**Figure 2:**
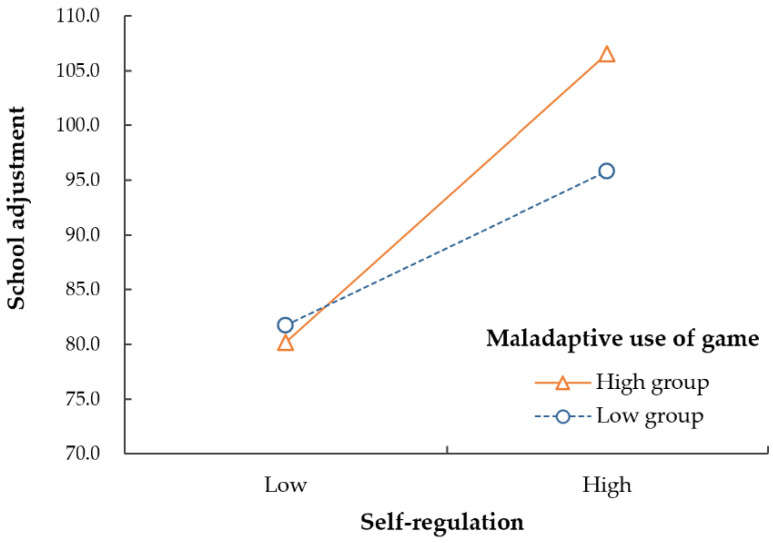
Results of the moderating effect of self-regulation.

The moderating effects between maladaptive game use and self-regulation are illustrated in Figure 2; the higher the adolescents’ self-regulation, the stronger the effect on school adjustment in the group with highly maladaptive game use.

To summarize, adaptive game use had a significant positive effect on school adjustment, and adolescent self-regulation improved school adjustment more strongly in the group with maladaptive game use.

## 4. Discussion

This study examined the moderating effect of self-regulation on the impact of adolescent gaming behaviors on school adjustment. To this end, data from 359 adolescents were analyzed using hierarchical regression analysis. The discussion below is based on the findings of this study.

Adolescents’ adaptive gaming behavior is a strong predictor of their school adjustment. When we examined the differences in school adjustment across game types categorized by adaptive and maladaptive use, we found that even high maladaptive game use was not associated with significantly lower school adjustment when combined with high adaptive use of games. This is similar to previous studies that have revealed that the adaptive-use group has lower academic stress and better parent–peer relationships than the other groups [7]. These results suggest that the adaptive use of games is a positive factor that reduces stress and allows adolescents to enjoy a healthy leisure life [25], which is related to improving school adjustment. Previous research on children and adolescents has reported that in groups that play several online games, successful gaming experiences have a positive impact on school adjustment and life satisfaction, albeit with some limitations [65]. This is a result of leveraging the positive aspects of gaming behavior. This suggests that adolescents should be educated to use games adaptively, rather than unconditionally, to regulate their gaming behaviors. Programs such as game literacy education, coding education, and game-culture family camps are being implemented in Korea to help adolescents use games adaptively, and it is necessary to expand them [66].

However, adolescents’ maladaptive use of games negatively affects school adjustment. This finding is similar to several previous studies that revealed that the addictive use of games negatively affects school adjustment [67,68,69,70,71]. Adolescents’ maladaptive use of gaming can lead to an inability to maintain daily routines, which, in turn, can lead to poor school performance. Therefore, it is important to reduce the maladaptive use of games among adolescents. Prevention policies and research addressing psychological and environmental domains have been proposed as ways to reduce maladaptive gaming use among youth. To reduce the maladaptive use of gaming among youth, the South Korean government implemented the “shutdown system,” which prohibited youth from playing games late at night. However, owing to an increase in mobile games, this has been abolished because of the loss of temporal and spatial boundaries. This suggests that government-enforced bans on gaming behavior are not effective, and that self-regulation is more important for youth to reduce gaming behavior on their own. Furthermore, attempts were made to develop and verify the effectiveness of game prevention education programs in preventing game addiction [72], and the need to present differentiated prevention programs according to the developmental stages of game users has been suggested [73]. Previous studies have reported resilience [74,75,76], self-efficacy [31], and positive psychological capital [77] as factors that prevent gaming addiction among adolescents. These factors suggest the importance of individual psychological functioning in relation to gaming behavior, and we confirmed the importance of self-regulatory capacity in modulating psychological functioning in adolescent gaming behaviors. In the present study, the negative effect of maladaptive gaming on school adjustment was not statistically significant when self-regulation was included. This suggests that self-regulation plays a significant role in maladaptive gaming behavior.

The results revealed a significant moderating effect of self-regulation on the negative impact of maladaptive adolescent gaming use on school adjustment. This finding highlights the importance of self-regulation in reducing maladaptive gaming among adolescents. Similar to the findings of this study, previous studies have also reported that game users’ self-regulatory tendencies have a moderating effect on excessive games [9]. Self-regulation is a higher-order concept of self-control [78], and failures in self-regulation have been linked to eating disorders, drugs, and gambling addictions [79]. Furthermore, high self-regulation is a protective factor against gaming over-engagement [80,81]. This suggests that self-regulation is an important protective factor against maladaptive gaming use among adolescents. In particular, the effect of self-regulation on school adjustment was stronger among adolescents with maladaptive gaming use, suggesting that self-regulation may be an alternative to maladaptive gaming.

However, it is important to note that risky-use adolescents, who have high levels of both adaptive and maladaptive use of games, mistakenly believe they are using games adaptively. This is because they are unaware of their maladaptive use, and they may be less willing to adopt a therapeutic approach to their gaming behavior, which can be even more problematic. They may also be more resistant to being asked to reduce their gaming or intervene in over-engaged gaming behaviors because they are usually well adjusted to school. However, it is imperative to recognize that the risky-use group’s self-regulation was significantly lower than that of the adaptive-use group in this study. Thus, the risky-use group can transition into the problematic-use group at any time. Therefore, it is necessary to improve self-regulatory skills to prevent risky-use groups from transitioning into problematic-use groups. Adolescents may resist external control owing to their developmental characteristics, as it is important for them to utilize apps that manage their smartphone usage history or identify their own usage habits for self-regulation [82]. Previous studies have suggested programs to improve adolescents’ self-regulation skills, such as setting goals for self-regulation; keeping records; and maintaining self-observation, self-evaluation, and self-reinforcement [83]. Additionally, stress is a major factor hindering adolescent self-regulation [84], and meditation therapies such as mindfulness group programs are reported to have positive effects on improving adolescent self-regulation [85]. Improving adolescents’ self-regulatory skills is an important protective factor against the progression of risky-use groups to problematic-use groups and is expected to serve as a stepping stone for the prevention of problematic-use groups progressing to risky-use groups.

To summarize the implications of this study, we found that the adaptive use of games supported adolescents’ school adjustment, and self-regulation was critical in reducing the negative impact of the maladaptive use of games on school adjustment. Furthermore, the risky-use group had a lower self-perception of the maladaptive use of games, indicating the requirement for active counseling interventions.

This study has some limitations. These are provided below along with the suggestions for future research.

First, this study did not measure gaming behaviors specific to the types of games teenagers frequently played. Future studies should measure gaming behaviors specific to different types of games, such as puzzle games, shooters, and role-playing games, commonly played by adolescents.

Second, in the present study, school adjustment was not significantly different between the risky-use group, which had both high maladaptive and adaptive use of games, and the adaptive-use group. Future research should investigate and propose psychological interventions for risky users who are unaware of the maladaptive use of games.

Third, this study examined gaming behaviors among middle and high school students and did not include the gaming behaviors of elementary and college students. In future research, it is necessary to conduct a comparative study of gaming behaviors and self-regulatory competence among college students who have recently experienced increased job stress and elementary school students who have experienced increased academic stress.

## 5. Conclusions

It was confirmed that the adaptive use rather than maladaptive use of games was an important predictor of adolescents’ school adaptation. Adolescents’ self-regulatory competency significantly moderated the effect of the maladaptive use of games on school adaptation. Individuals in the risky-use group, with high levels of both adaptive and maladaptive use of games, require active psychological intervention because they are unaware of the problems with their gaming behavior and lack the willingness to engage in voluntary therapeutic counseling.

## Figures and Tables

**Table 1:** Descriptive statistics and correlation coefficients of research variables (*n* = 359).

Variables	Maladaptive Use of Games	Self-Regulation	School Adjustment	M ± SD
Adaptive use of games	0.40 ***	0.15 **	0.23 ***	32.05 ± 14.84
Maladaptive use of games	1.00	−0.31 ***	−0.20 ***	12.15 ± 12.62
Self-regulation		1.00	0.54 ***	55.06 ± 9.12
School adjustment			1.00	88.44 ± 14.80

** *p* < 0.01, *** *p* < 0.001.

**Table 2:** Differences in self-regulation and school adjustment by gaming behavior group (*n* = 359).

Gaming Behavior Group	Self-Regulation		School Adjustment	
	M ± SD	F (*p*)	M ± SD	F (*p*)
Adaptive-use group ^(a)^	57.22 ± 9.09	8.75 (<0.001)	92.40 ± 15.01	9.50 (<0.001)
General-use group ^(b)^	54.38 ± 8.97	a > b > d	86.33 ± 13.78	a > b > d
Risky-use group ^(c)^	52.04 ± 5.19	a > c	89.43 ± 13.94	c > d
Problematic-use group ^(d)^	43.25 ± 9.42		69.63 ± 15.51	

^abcd^ Alphabets represent each group.

**Table 3:** Moderating effects of self-regulation in the effects of gaming behavior on school adjustment (*n* = 359).

Variables (Reference)	Model 1		Model 2		Model 3	
	B	t (*p*)	B	t (*p*)	B	t (*p*)
Gender (male)	2.25	1.42 (0.157)	2.06	1.51 (0.132)	2.34	1.72 (0.087)
Age	−0.40	−0.57 (0.556)	−0.13	−0.22 (0.830)	−0.24	−0.41 (0.682)
Adaptive use of games (low group) ^(a)^	7.40	4.59 (<0.001)	4.74	3.37 (0.001)	4.40	3.12 (0.002)
Maladaptive use of games (low group) ^(b)^	−6.54	−2.36 (0.019)	−0.81	−0.33 (0.741)	2.78	0.95 (0.343)
Self-regulation ^(c)^			0.83	11.19 (<0.001)	0.72	7.38 (<0.001)
Interaction 1 ^(a×c)^					0.17	1.16 (0.247)
Interaction 2 ^(b×c)^					0.67	2.12 (0.035)
R^2^ (F, *p*)	0.068 (F = 6.42, *p* < 0.001)		0.312 (F = 31.99, *p* < 0.001)		0.322 (F = 23.85, *p* < 0.001)	

^abc^ Alphabets represent each variable.

## Data Availability

The data presented in this study are not available elsewhere.
